# Diagnostic performances of *Schistosoma haematobium* and *Schistosoma mansoni* recombinant proteins, peptides and chimeric proteins antibody based tests. Systematic scoping review

**DOI:** 10.1371/journal.pone.0282233

**Published:** 2023-03-02

**Authors:** Arthur Vengesai, Victor Muleya, Herald Midzi, Tryphine Vimbai Tinago, Isaac Chipako, Marble Manuwa, Thajasvarie Naicker, Takafira Mduluza

**Affiliations:** 1 Department of Biochemistry, Faculty of Medicine and Health Sciences, Midlands State University, Gweru, Zimbabwe; 2 Department of Biochemistry, University of Zimbabwe, Mt Pleasant, Harare, Zimbabwe; 3 Aravas Pharmaceuticals Pvt LTD, Prospect Industrial Area, Harare, Zimbabwe; 4 Discipline of Optics and Imaging, Doris Duke Medical Research Institute, College of Health Sciences, University of KwaZulu-Natal, Durban, South Africa; George Washington University, UNITED STATES

## Abstract

**Background:**

Traditional diagnostic tests for schistosome infections are suboptimal, particularly when the parasite burden is low. In the present review we sought to identify recombinant proteins, peptides, and chimeric proteins with potential to be used as sensitive and specific diagnostic tools for schistosomiasis.

**Methods:**

The review was guided by PRISMA-ScR guidelines, Arksey and O’Malley’s framework, and guidelines from the Joanna Briggs Institute. Five databases were searched: Cochrane library, PubMed, EMBASE, PsycInfo and CINAHL, alongside preprints. Identified literature were assessed by two reviewers for inclusion. A narrative summary was used to interpret the tabulated results.

**Results:**

Diagnostic performances were reported as specificities, sensitivities, and AUC. The AUC for *S*. *haematobium* recombinant antigens ranged from 0.65 to 0.98, and 0.69 to 0.96 for urine IgG ELISA. *S*. *mansoni* recombinant antigens had sensitivities ranging from 65.3% to 100% and specificities ranging from 57.4% to 100%. Except for 4 peptides which had poor diagnostic performances, most peptides had sensitivities ranging from 67.71% to 96.15% and specificities ranging from 69.23% to 100%. *S*. *mansoni* chimeric protein was reported to have a sensitivity of 86.8% and a specificity of 94.2%.

**Conclusion:**

The tetraspanin CD63 antigen had the best diagnostic performance for *S*. *haematobium*. The tetraspanin CD63 antigen Serum IgG POC-ICTs had a sensitivity of 89% and a specificity of 100%. Peptide Smp_150390.1 (216–230) serum based IgG ELISA had the best diagnostic performance for *S*. *mansoni* with a sensitivity of 96.15% and a specificity of 100%. Peptides were reported to demonstrate good to excellent diagnostic performances. *S*. *mansoni* multi-peptide chimeric protein further improved the diagnostic accuracy of synthetic peptides. Together with the advantages associated with urine sampling technique, we recommend development of multi-peptide chimeric proteins urine based point of care tools.

## Introduction

Schistosomiasis, otherwise known as bilharzia or the snail fever, is the second most significant tropical parasitic disease after malaria [1]. Despite significant global control efforts schistosomiasis continues to pose a major public health burden [2]. Schistosomiasis affects almost 240 million people worldwide, spanning least 78 countries and affecting more than 700 million people within endemic areas [3, 4]. Approximately 90% of the global cases occur in sub-Saharan Africa were approximately 300 000 deaths are estimated emanate from *S*. *mansoni* and *S*. *haemat*obium infections [5]. Globally there are about 436 million people at risk of infection, with 112 million people infected with *S*. *haematobium*. *S*. *mansoni* is the main cause of intestinal schistosomiasis in Sub Saharan Africa places 393 million people at risk of infection and infects 54 million people globally [3, 4].

One of the major obstacles to sustained disease control and eradication is due to inadequate diagnostic approaches that are highly sensitive, inexpensive, rapid, and that can be utilized at the point of care [2, 6]. Sensitive diagnostic approaches plays a vital role in, generating data that influence decisions on individual and community treatment, assessment of morbidity and evaluation of chemotherapy and other control measures [7, 8].

Traditional parasitological methods (Kato Katz technique and urine filtration) show low sensitivity, especially in infections of low intensity that are most likely encountered in interruption of transmission scenarios [9]. Moreover, many light infections are missed due to absence of eggs in urine and stool specimens [10–13]. Even in many high-endemic settings, the average infection intensity is often low, and microscopy alone may thus easily miss a considerable number of infections [14]. In addition, one of the caveats of microscopy as a diagnostic tool is that it is labour-intensive and time-consuming [15]. The failure of the traditional egg detection methods emphasises the need for more sensitive diagnostic methods to effectively control and monitor schistosomiasis.

While alternative methods for schistosomiasis diagnosis are available, these methods have shortcomings. One of the alternative diagnostic procedures is the PCR-based method, which confers high specificity and sensitivity in detecting schistosome infections. However, the method is expensive and requires skilled personnel which are not readily available in remote rural settings [7, 16]. The point-of-care circulating cathodic antigen test is considerably more sensitive than the Kato-Katz technique but shows low sensitivity when compared to ELISA in low infection intensity cases [17, 18]. Moreover, it has been noted that the POC-CCA tends to give false negative results for light intensity infections [19]. On the other hand, serological based tests such as ELISAs using crude antigens such as soluble egg antigens, increases diagnostic accuracy in low burden areas. However, crude antigens are of limited value in endemic regions because of high costs of production, low-specificity and they often cross-react with other helminths [20].

### Review aims and objectives

In this scoping review, we sought to identify recombinant proteins, peptides and chimeric proteins with potential applications in diagnosis of *S*. *haematobium* and *S*. *mansoni*. In line with the WHO Department of Control of Neglected Tropical Diseases Diagnostic Technical Advisory Group, we aim to identify recombinant proteins, peptides and chimeric proteins that can be used to develop sensitive, point of care diagnostics for *S*. *haematobium* and *S*. *mansoni* in different prevalence settings that can be used for surveillance and transmission assessment.

## Methods

This review was conducted using PRISMA-ScR guidelines (S1 Table) to search and select relevant studies for inclusion in the scoping review [21]. The methodological framework proposed by Arksey and O’Malley (2005) and further refined by the Joanna Briggs Institute [22, 23] was used to develop the protocol and guide data extraction. Our methodology was also based upon previous reviews by our group [24, 25] in conjunction with other relevant scoping reviews [26–28]. As a scoping review is a continual process, the methodology was continuously revised as the review progressed.

### Search strategy

#### Electronic searches

The Cochrane library, PubMed, EMBASE, PsycInfo and CINAHL databases were systematically searched for relevant articles without any language restrictions. To maximise relevance of the findings, literature search included the time frame of January 2000 to February 2022. The databases were searched using variations and combinations of the following keywords: recombinant proteins, peptides, and schistosomiasis. The search strategy and results for all databases are S1 File.

#### Searching other resources

Preprint databases MedRxiv and BioRxiv were also searched. The reference lists of relevant reviews and studies and websites of the World Health Organization (WHO), the Schistosomiasis Control Initiative (SCI), and the Schistosomiasis Consortium for Operational Research and Evaluation (SCORE) were also screened for additional articles.

### Criteria for considering studies for this review (inclusion and exclusion criteria)

Cross-sectional, longitudinal, case–control, diagnostic accuracy studies and clinical trials, looking at human *S*. *haematobium* and/or *S*. *mansoni* infections and recombinant antigens and/or peptides diagnostic serological assays were included in the scoping review. In cases where there were different subpopulations, the overall diagnostic accuracy was considered. On protein microarrays, antigens able to effectively discriminate between schistosomiasis infected and non-infected populations using sera or urine were considered.

Studies were excluded if:

- Combinations of recombinant antigens
- Full text and abstract were both unavailable or only the abstract was available but did not convey the needed data.
- Conference abstract
- Human *Schistosoma* infection was not investigated.
- Data from the *Schistosoma*-only infections could not be extracted.
- Narrative reviews.
- Circulating anodic (CAA) and cathodic (CCA) antigen or other commercially available antigens were being investigated
- Animal models

### Review process and data charting

One reviewer independently assessed the eligibility of the abstracts, downloading relevant articles into Mendeley reference manager, whilst removing duplicate articles. After removing duplicates shortlisted articles were screened for inclusion through full-text analysis. Two reviewers independently assessed the eligibility of the full text articles. After the articles were selected, two authors extracted and recorded the data from each study according to a predefined data extraction excel spreadsheet form. A third reviewer was consulted to resolve disagreements. The extracted data included author, year of publication, DOI, study design, aim and study domain, country where study was conducted, schistosome species, serological assay type, sample characteristics, negative control, reference standard, antigen type, antibody type, index test and diagnosis accuracy of antigen (sensitivity and specificity).

### Methodological quality appraisal and data synthesis

Methodological quality or risk of bias of the included articles was not appraised, which is consistent with guidance on scoping review conduct [22]. Descriptive numerical analysis was performed and a narrative summary was used to interpret the tabulated results and describe how they relate to the review’s aim and questions [29].

## Results and discussion

### Identification of potential studies

**Fig 1:**
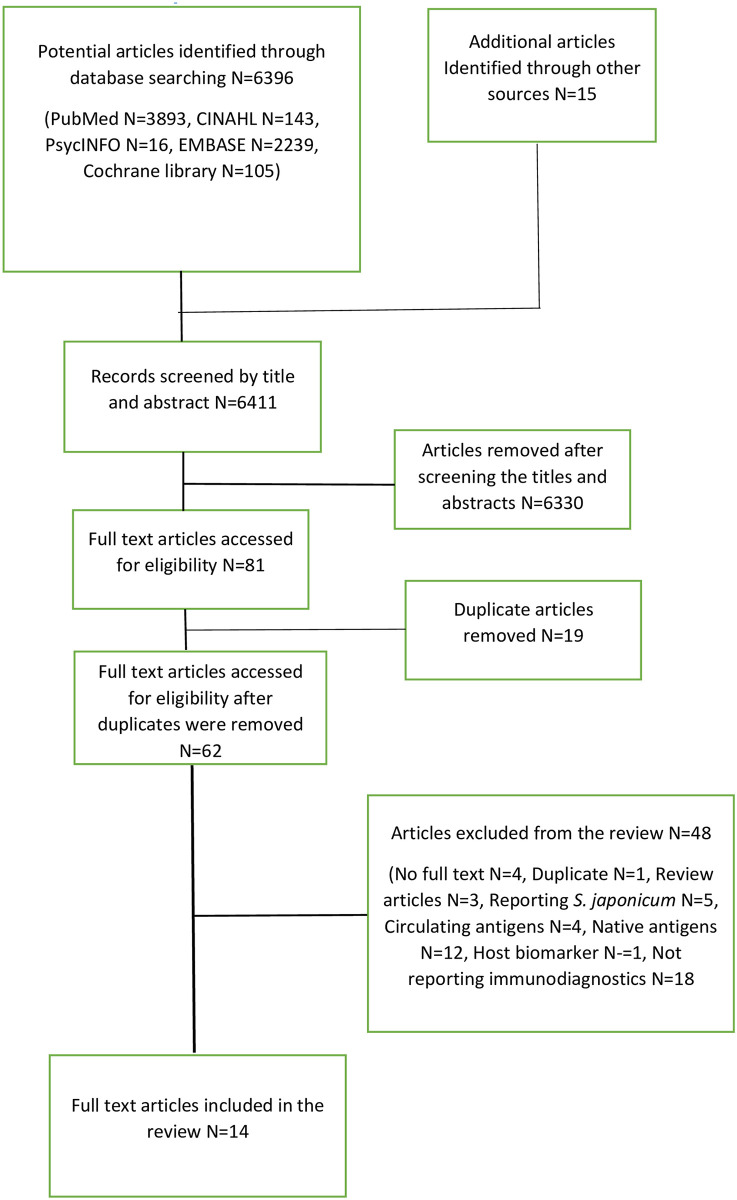
Flow chart of the studies identification and selection process.

Electronic searches of five databases Pubmed (n = 3893) CINAHL (n = 1629), Cochrane library (n = 143), PsycINFO (n = 16) and EMBASE (n = 2239) yielded 6396 potential articles for inclusion in the review. Additional articles (n = 15) identified through other sources led to a total of 6411 titles and abstracts eligible for screening. A total of 62 full text articles were screened for eligibility after the removal of duplicate articles (n = 19) and irrelevant articles (n = 6330). Full text screening led to a total of 14 articles that were included in the systematic scoping review. Fig 1 is demonstrating the flow chart of the studies identification and selection process. A total 48 studies were excluded from the review after full text screening and reasons for exclusion are given Fig 1 and S2 Table.

### Characteristics of the included articles

All the articles included in the review were non- randomized controlled trials. Among the articles selected, one was a preprint [30]. The general characteristics of the articles included in the scoping review are shown in Table 1. The articles included were from 6 countries Brazil (n- = 7), Australia (n = 2), Kenya (n = 2), United Kingdom (n = 1), Spain (n = 1), and Italy (n = 1). All the included studies were investigating human schistosome immunodiagnostics, apart from one study by Mekonnen and colleagues [31] who also used a mouse model to investigate schistosome immunodiagnostics. Most of the articles (71.42%) included in the review sought to identify peptides and recombinant proteins with potential applications in the detection of *S*. *mansoni*. Only 28.58% (n = 4) sought to identify peptides and recombinant proteins with potential applications in the detection of *S*. *haematobium*. Two articles investigated immunodiagnostics of both species. Although IgM has better diagnostic features than IgG responses [32], only one study, by Mambelli and colleagues, investigated IgM based serological test [33]. Like most available assays most of studies included in the review detected IgG only which have been reported to be more specific than IgM [34]. The study by Mambelli and colleagues investigated both IgG and IgM [33].

**Table 1:** General characteristics of the studies included in the review.

Index test	Species	Study Population	Sample characteristic	Control	Antibody type	Reference test	Geographic location	Year	Reference
Protein microarray, ELISA, and POC-ICTs	*S*. *haematobium*	Humans	Serum/plasma and Urine	Specificity control sera	IgG	Egg detection	Australia	2021	Mark S Pearson [35]
ELISA	*S*. *haematobium*	Humans and mice	Urine	Healthy negative control sera	IgG	Egg detection	Australia	2022	Gebeyaw G. Mekonnen [31]
ELISA	*S*. *mansoni*	Humans	Serum/plasma	Specificity control sera	IgG	Egg detection	Brazil	2008	E. De Oliveira [36]
ELISA	*S*. *mansoni*	Humans	Serum/plasma	Healthy negative control sera	IgG	Egg detection	Brazil	2017	Gardenia B. F. Carvalho [16]
ELISA	*S*. *mansoni*	Humans	Serum/plasma	Healthy negative control sera	IgG	Egg detection	Brazil	2017	Marcelo D. Lopes [37]
ELISA	*S*. *mansoni*	Humans	Serum/plasma	Healthy negative control sera	IgG	Egg detection	Brazil	2019	Vanessa Silva-Moraes [38]
ELISA	*S*. *mansoni*	Humans	Serum/plasma	Healthy negative control sera	IgG and IgM	Egg detection	Brazil	2020	Mambellia, F.S. [33]
DELFIA and Protein microarray immunoassays	*S*. *mansoni*	Humans	Serum/plasma	Healthy negative control sera	IgG	Bordier Affinity Products CH and Egg detection.	Italy	2021	Stefano De Benedetti [39]
ELISA	*S*. *mansoni*	Humans	Serum/plasma	Healthy negative control sera	IgG	Egg detection	Kenya	2021	Mio Tanaka [17]
ELISA	*S*. *mansoni*	Humans	Serum/plasma	Specificity control sera	IgG	Egg detection	United Kindom	2010	Bahaa El Deen Wade El Aswada [20]
Luminex multiplex immunoassay	*S*. *mansoni S*. *haematobium*	Humans	Serum/plasma	Healthy negative control sera	IgG	Egg detection	Kenya	2015	Tanigawa [40]
Luminex multiplex immunoassay	*S*. *mansoni S*. *haematobium*	Humans	Serum/plasma	Healthy negative control sera	IgG	Egg detection	Spain	2022 (Preprint)	Rebeca Santano [30]
ELISA	*S*.*mansoni*	Humans	Serum/plasma	Specificity control sera	IgG	Egg detection	Brazil	2003	E. Makarova [41]
ELISA and western blot	*S*.*mansoni*	Humans	Serum/plasma	Specificity control sera	IgG	Egg detection	Brazil	2005	E. Makarova [42]

Dissociation-Enhanced Lanthanide Fluoroscence ImmunoAssays (DELFIA)

Point of care immunochromatographic tests (POC-ICT)

#### Negative controls

Negative controls are primarily used to evaluate the specificity of a test. In other words, negative controls are used to correctly identify individuals without parasitic infections (the true negatives) while minimizing false positive results as determined by the reference method [43, 44]. Negative controls generally consist of tissues (including blood) where the target proteins for instance antibodies are known to be absent [34]. In the current review, all negative sera were obtained from schistosomiasis unexposed individuals without *Schistosoma* infections and without a history of contact with contaminated water. Negative controls can be differentiated into normal negative controls (from healthy individuals) or specificity controls from individuals (from individuals infected with other parasites) [35, 41, 42, 45]. Specificity controls are important to determine whether a test can designate an individual who do not have the target infection as negative. In the present review most of the studies (N = 9) used normal controls as the negative control and only 5 studies used specificity controls as the negative control.

#### Antibody-detection tests and antigen-detection tests

Studies included in the present review used antibody-detection tests that have been merited with high sensitivity. These antibody detection tests included ELISA (used in 80% of the studies) and point of care immunochromatographic tests (POC-ICT) (used in one study [35]) which are easy to perform [46]. These antibody-detection tests are indirect tests that look for schistosome antibodies in body fluids such as serum, plasma and urine that are produced in response to infections by schistosome species [47, 48]. Ideally, a serological test must use a single and specific schistosome antigen that is recognized by the immune response of all schistosome infected humans. The response of the test should be approximately proportional to the worm burden and would rapidly fall towards zero when the stimulus provided by infection was eliminated by praziquantel chemotherapy [32]. However, the antibody-detection tests included in the review have been found to be disadvantageous in that they have low specificity and do not accurately reflect the presence of active infections particularly in endemic areas. Antibodies may persist systemically for many months to years after successful treatment. This implies that antibody detection tests are unable to differentiate between acute or past infections nor discrimination between persisting antibodies and reinfection. [46, 49, 50]. Additional antibody-detection tests do not reflect infection intensity [46]. They are beneficial in patients with infrequent exposure to schistosome cercariae, such as tourists visiting an endemic area [32]. Fortunately, there may be certain antigens to which specific antibody isotype subclasses such as IgG_4_, disappear more rapidly [9]. These antibodies can be targeted to circumvent the limitation associated with persisting antibodies.

Antigen-detection tests are a better alternative in the diagnosis of schistosome infections as they differentiate between active and past infections, because the circulating antigens are present only when there is active infection [46, 48]. Since circulating antigens are released from living worms, antigen levels may correlate directly with parasite load, unlike microscopy and antibody-detection tests. This may make antigen-detection tests useful in monitoring the dynamics of worm burdens and clearance of worms after treatment [48]. Furthermore, antigen-detection tests allow for non-invasive diagnosis as antigens are cleared from the human circulation via the kidneys and can be detected in urine [51]. However, it is noteworthy to mention that antigen-detection tests exhibit low sensitivity [46].

#### Invasive venepuncture and non-invasive urine sampling

Urine sampling is a generally painless, more convenient, less expensive, and non-invasive technique hence of low risk. Urine sampling negates the need for well-trained personnel, is better accepted by patients and is usually obtained immediately upon request [46, 52]. Pearson and colleagues furthermore reported that urine IgG signal intensity was significantly correlated with schistosome infection intensity but reported no correlation between infection intensity and serum IgG signal intensity [35]. Despite the significant advantages of the ease and non-invasive urine sampling only two studies included in this review used this technique. Henceforth, most studies 93% (n = 14) included in the review involved the invasive collection of blood via venepuncture. Apart from the obvious risk of infection associated with venepuncture, the technique is painful, onerous, and highly invasive for children. Venepuncture is a limiting technique because it requires specific equipment and well-trained personnel seldom available in schistosomiasis endemic rural areas. In addition, venepuncture is not widely accepted especially by children and certain ethnic and religious groups [53, 54]. Also, the serum and/or plasma samples utilized in the studies reported in the review requires blood separation which can be difficult in field conditions as it requires a centrifuge [54]. However, it should be noted that the increased amount of antibodies in serum maximise the success of pilot tests, which could then be optimized for use with urine [35]. An alternative could be utilization of finger prick blood sampling based tests which are easier to use and easy to develop it into a point of care test or self-use test. However, tests are less sensitivity compared to tests based on venepuncture sampling [55]. Alternatively, saliva may be used as a non-invasive alternative to serum. Zhou and colleagues (2009) reported the use of recombinant Sj13 as an antigenic target in an IgG-ELISA using saliva and showed comparable performance to SjSWA-IgG ELISA in serum and a low cross-reactivity in *S*. *japonicum* endemic areas [56].

### *S*. *haematobium* and *S*. *mansoni* recombinant antigens

For narrative review, recombinant antigens were grouped into 11 schistosome protein families. serine protease inhibitors (SERPIN), Pur DNA binding protein, MEC 2, tetraspanin, peptidases, ferritins and haemoglobinase for *S*. *haematobium* (Table 2). Chaperone, micro-exon gene (MEGs) proteins, saposin-like proteins (SAPLIP) and calreticulin were identified for *S*. *mansoni* (Table 3). The diagnostic performance of *S*. *haematobium* recombinant antigens measured using area under the curve (AUC) ranged from 0.65 (95% CI 0.54–0.76) [ferritin heavy polypeptide Urine IgG protein microarray] to 0.98 (95% CI 0.95–1.00) [Sh-TSP2 tetraspanin serum IgG ELISA]. The non-evasive relatively easy to use urine IgG ELISA AUC ranged from 0.69 (95% CI 0.62–0.77) to 0.96 (95% CI 0.93–0.99). *S*. *mansoni* recombinant antigens had sensitivities ranging from 65.3% (95% CI 55.0–76.6%) [CCD60071 SERPIN IgG ELISA] to 100% [RP26 SAPLIP serum IgG western blot] and specificities ranging from 57.4% (95% CI 55.0–76.6%) [AAB1008 RP26 SAPLIP serum IgG ELISA] to 100% [AAB1008 RP26 SAPLIP serum IgG western blot].

**Table 2:** Diagnostic performance *S*. *haematobium* recombinant antigens antibody detection tests.

Antigen (accession)	Description	Protein family	Serological assay	Sensitivity	Specificity	AUC& (95% CI)	Reference
MS3_10385	neuroserpin**+**	Serpin [35]	Serum IgG protein microarray	-	-	0.88 (0.83–0.92)	Mark S Pearson [35]
MS3_10186	IPSE	None predicted [57]	Serum IgG protein microarray	-	-	0.88 (0.83–0.92)	
MS3_06193	PUR-alpha-like protein	PUR DNA binding protein (uniport.org)	Serum IgG protein microarray	-	-	0.71 (0.64–0.79)	
MS3_01466	band 7 protein	Mec 2 (uniport.org)	Serum IgG protein microarray	-	-	0.69 (0.62–0.75)	
MS3_05950	16 kDa calcium-binding protein	None predicted [57]	Serum IgG protein microarray	-	-	0.76 (0.70–0.82)	
MS3_09198	CD63 antigen+	Tetraspanin (uniport.org])	Serum IgG protein microarray	-	-	0.79 (0.73–0.85)	
MS3_09779	cathepsin B-like peptidase (C01 family) +	Peptidase C1 (uniport.org)	Serum IgG protein microarray	-	-	0.84 (0.76–0.93)	
MS3_07972	ferritin, heavy polypeptide 1+	Ferritin (uniport.org)	Serum IgG protein microarray	-	-	0.86 (0.80–0.92)	
MS3_09207	hemoglobinase (C13 family) +	Peptidase C13 (uniport.org)	Serum IgG protein microarray	-	-	0.78 (0.71–0.84)	
MS3_01370	CD63 antigen+	Tetraspanin [35]	Serum IgG protein microarray	-	-	0.78 (0.71–0.84)	
MS3_10385	neuroserpin	Serpin [35]	Urine IgG protein microarray	-	-	0.93 (0.85–1.00)	
MS3_10186	IPSE	None predicted [57]	Urine IgG protein microarray	-	-	0.88 (0.80–0.97)	
MS3_06193	PUR-alpha-like protein	PUR DNA binding (uniport.org)	Urine IgG protein microarray	-	-	0.83 (0.75–0.91)	
MS3_01466	band 7 protein	Mec 2 (uniport.org)	Urine IgG protein microarray	-	-	0.75 (0.66–0.84)	
MS3_05950	16 kDa calcium-binding protein	None predicted (uniport.org)	Urine IgG protein microarray	-	-	0.72 (0.62–0.82)	
MS3_09198	CD63 antigen+	Tetraspain [35]	Urine IgG protein microarray	-	-	0.83 (0.73–0.92)	
MS3_09779	cathepsin B-like peptidase (C01 family) +	Peptidase C1 (uniport.org)	Urine IgG protein microarray	-	-	0.66 (0.55–0.76)	
MS3_07972	ferritin, heavy polypeptide 1+	Ferritin (uniport.org)	Urine IgG protein microarray	-	-	0.65 (0.54–0.76)	
MS3_09207	hemoglobinase (C13 family) +	Haemoglobinase (uniport.org)	Urine IgG protein microarray	-	-	0.68 (0.57–0.78)	
MS3_01370	CD63 antigen	Tetraspanin (uniport.org)	Urine IgG protein microarray	-	-	0.66 (0.56–0.77)	
MS3_10385	Neuroserpin+	Serpin [35]	Serum IgG ELISA	-	-	0.80 (0.70–0.91)	
MS3_10186	IPSE	Tetraspsnin [35]	Serum IgG ELISA	-	-	0.88 (0.82–0.94)	
MS3_09198	CD63 antigen+	Tetraspanin (uniport.org)	Serum IgG ELISA	-	-	0.82 (0.74–0.91)	
MS3_01370	CD63 antigen	Tetraspsnin [35]	Serum IgG ELISA	-	-	0.93 (0.89–0.97)	
*Sh*-TSP2		Tetraspanin [31]	Serum IgG ELISA	-	-	0.98 (0.95–1.00)	
MS3_10385	Neuroserpin+	Serpin [35]	Urine IgG ELISA	-	-	0.78 (0.71–0.86)	
MS3_10186	IPSE	Tetraspanin [35]	Urine IgG ELISA	-	-	0.69 (0.62–0.77)	
MS3_09198	CD63 antigen+	Tetraspanin (uniport.org)	Urine IgG ELISA	-	-	0.78 (0.67–0.88)	
MS3_01370	CD63 antigen	Tetraspanin [35]	Urine IgG ELISA	-	-	0.81 (0.72–0.89)	
Sh-TSP2	Tetraspanin	Tetraspanin [31]	Urine IgG ELISA	-	-	0.96 (0.93–0.99)	
MS3_01370	CD63 antigen	Tetraspanin [35]	Serum IgG POC-ICTs	89%	100%	-	
Sh-TSP2	Tetraspanins	Tetraspanin [31]	Serum IgG POC-ICTs	75%	100%	-	
Sh-TPS-4	Tetraspanins	Tetraspanin [31]	Urine IgG ELISA	-	-	0.87	Gebeyaw G. Mekonnen [31]
Sh-TPS-5	Tetraspanins	Tetraspanin [31]	Urine IgG ELISA	-	-	0.93	
Sh-TPS-18	Tetraspanins	Tetraspanin [31]	Urine IgG ELISA	-	-	0.88	
AAA19730	Serine protease inhibitor	Serpin [58]	Serum IgG Luminex immunoassay	92.5%	90%	0.947	Tanigawa [40]
	Sm 25		Serum IgG Luminex immunoassay	-	-	0.711	Rebeca Santano [30]

Serine protease inhibitor (SERPIN)

Point of care immunochromatographic tests (POC-ICT)

**Table 3:** Diagnostic performance of *S*. *mansoni* recombinant antigens antibody detection tests.

Antigen (accession)	Description	Protein family	Serological assay	Sensitivity	Specificity	AUC& (95% CI)	Reference
Smp_049250.1	Major egg antigen	Cheparone [38, 59]	Serum IgG ELISA	87.1 (95% CI 78.55–93.15%)	89.09% (77.75–95.89%)	-	Vanessa Silva-Moraes [38]
Smp_138060—CCD60408.1	MEG 3.2	MEG 3 [33]	Serum IgM ELISA	90%	70%	-	Mambellia, F.S. [33]
Smp_138060—CCD60408.1	MEG 3.2	MEG 3 [33]	Serum IgG ELISA	90%	83%	-	
Smp_138090—CAZ30619.1)	MEG 3.4	MEG 3 [33]	Serum IgM ELISA	75%	90%	-	
SmSPI	Serine protease inhibitor	Serpin [60]	Serum IgG protein microarray	91.7%	93.3%	-	Stefano De Benedetti [39]
SmSPI	Serine protease inhibitor	Serpin [17, 60]	Serum IgG DELFIA	83.7%	61.4%	-	
AAB81008	RP 26	SAPLIP [40, 61]	Serum IgG ELISA	74.5% (95% CI 66.7–82.8%)	57.4% (95% CI 47.2–67.2)	-	Mio Tanaka [17]
CCD60071	Serine protease inhibitor	Serpin [17, 60]	Serum IgG ELISA	65.3% (95% CI 55.0–74.6%)	62.4% (52.2–71.8%)	-	
Sm CRT	Calreticulin	Calreticulin [20]	Serum IgG ELISA	89.7%	100%	-	Bahaa El Deen Wade El Aswada [20]
AAB81008	RP 26 (Sm 22.3)	SAPLIP [40]	Serum IgG Luminex immunoassay	67.8%	89.5%	0.833	Tanigawa [40]
CCD60071	Serine protease inhibitor	Serpin [17, 60]	Serum IgG Luminex immunoassay	89.4%	81.5%	0.888	
	Major egg antigen (ME)	Cheparone [38, 59]	Serum IgG Luminex immunoassay	-	-	0.746	Rebeca Santano [30]
	Sm 25		Serum IgG Luminex immunoassay	-	-	0.741	
	RP 26	SAPLIP [17]	Serum IgG western blot	100%	100%	-	E. Makarova [41]
	RP 26	SAPLIP [17]	Serum IgG western blot	89%	-	-	E. Makarova [42]

Dissociation-Enhanced Lanthanide Fluoroscence ImmunoAssays (DELFIA)

Saposin-like proteins (SAPLIP)

Serine protease inhibitor (SERPIN)

Micro-exon gene (MEGs)

#### Saposin-like proteins (SAPLIP)

Saposin-like proteins are a multi-gene family of *Schistosoma* species that are potential biomarkers for the immunodiagnosis of schistosome infections [6, 62]. SAPLIPs expressed in the gastrodermis are involved in lysis of ingested host red blood cells in the gut of the parasite. These SAPLIPs may be continuously released into the host circulatory system in worm vomitus, stimulating the host to produce a strong immune response [62, 63]. In the present review *S*. *mansoni* RP26 a member of the SAPLIP multi-gene family was reported to have sensitivities ranging from 67.80% [Serum IgG luminex immunoassay] to 100% [Serum IgG Western blot] and specificities ranging from 57.4% [Serum IgG ELISA] to 100% [Serum IgG Western blot] [17, 40]. It was furthermore noted that RP26 may be particularly useful in developing tools for monitoring progress of chemotherapy intervention by detecting new infections. RP26 is expressed at the cercarial, schistosomula and immature worms’ stages, and not in the egg stage. This suggests that it has potential to detect acute schistosome infections [40]. Tanigawa and colleagues indeed found that mean IgG reactivity to RP26 was significantly higher during acute schistosomiasis, compared to the negative reaction observed to sera from chronic schistosomiasis [40].

#### Serine protease inhibitors (SERPINS)

SERPINS are interesting immunodiagnostic targets, due to their presence at the parasite-host interface, where they are crucial for the adaptation and survival of the parasite to the host environment by modulating inflammatory response [17]. Amongst the studies that have investigated *S*. *haematobium*, SERPINS AUC ranged from 0.78 (95% CI 0.71–0.86) to 0.947. Urine IgG ELISA had the lowest diagnostic accuracy of 0.78 (95% CI 0.71–0.86) and serum IgG Luminex immunoassay had the highest diagnostic accuracy with an AUC of 0.947, a sensitivity of 92.50% and specificity of 90%. Amongst the studies that investigated *S*. *mansoni*, SERPINS sensitivity ranged from 65.3% (95 CI 55.0–74.6) [Serum IgG ELISA] to 91.70% [Serum IgG protein microarray] and specificity ranged from 61.40 [Serum IgG DEFIA] to 93.30% [Serum IgG protein microarray]. The SERPINS SmSPI and CCD60071 were reported to show species-specific diagnosis of *S*. *mansoni*. Antibodies raised against SmSPI *S*. *haematobium* homolog did not recognize *S*. *mansoni* SmSPI [17, 40]. However, despite *S*. *haematobium* AAA19730 SERPIN performing well for the diagnosis of *S*. *haematobium* it showed cross-reactivity with sera from infections caused by *S*. *mansoni* [40].

#### Tetraspanins

Tetraspanins, also known as the trans-membrane 4 super-family, comprise a collection of surface antigens that are widely expressed in the schistosome species [64]. These antigens are abundantly present in different parasite proteomes and could be potential diagnostic candidates due to their accessibility to the host immune system [31, 64]. In the present review of *S*. *haematobium*, tetraspanins were reported to have relatively good diagnostic performance. Their AUC ranged from 0.66 (95% CI 0.56–0.77) to 0.96 (95% CI 0.93–0.99). Urine IgG ELISA AUC ranged from 0.69 (95% CI 0.62–0.77) to 0.96 (95% 0.93–0.99). Urine based POC LFIA had a sensitivity that ranged from 75% to 89% with a specificity of 100% [31, 35].

#### Calreticulin

Pathogenic species of schistosome including *S*. *mansoni* and *S*. *haematobium* secrete and express calreticulin on their surface [20] making it a potential diagnostic candidate for schistosome species identification. Indeed, Aswada and colleagues reported that *S*. *mansoni* calreticulin serum based IgG ELISA had a sensitive of 89.70% and a specificity of 100% [20].

#### Micro-exon gene proteins

Micro-exon gene proteins are also potential candidates for diagnosis of schistosomiasis as they are highly expressed in schistosome life cycle that are associated with the host infection and immune evasion by schistosome species [33]. MEG-3.2 distinguished sera of *S*. *mansoni* infected individuals from non-infected ones, supporting its potential for the development of a MEG based diagnostic tool [33]. *S*. *mansoni* MEG, serum based IgM ELISA was reported to have sensitivities ranging from to 75% to 90% and specificities ranging from 70% to 90% and serum based IgG ELISA was reported to have a sensitivity of 90% and a specificity of 83% [33].

The antibody based tests described in this review have several advantages compared to the parasitological diagnosis. These include high sensitivity over parasitological methods, relatively easy to automation, large scale processing of samples and POC-ICT are less time consuming [6, 36, 65]. However, the antibody based tests also have several limitations. Schistosome-specific antibodies remain detectable for a long period after the infection has been cured. Consequently, measuring anti-schistosome antibody titres in serum may not discriminate between current and past infections [36, 65]. Cross reactivity between antigens from Schistosoma species and other co-endemic helminths such as soil transmitted helminths may occur due to shared antigenic epitopes [36, 65]. Antibody concentrations do not necessarily correlate with infection intensity [36]. In this review only SERPIN CCD60071 had a correlation with infection intensity [40]. This has clinical implications in confirmation of successful praziquantel treatment since specific antibodies may persist long after the worms have disappeared [36]. Lack of reproducibility between different batches of reagents may further hinder or delay implementation of new diagnostics [65].

### Peptides

One way to circumvent the problems associated with antibody based tests is the use of synthetic peptides which are well defined, easily produced in large amounts when required, highly pure with almost no batch-to-batch variation and they are very stable and cost-saving compared to the production of natural antigens in animal models or from *in vitro* culture [24, 66, 67].

The use of synthetic peptides composed of species-specific and conserved epitopes have a potential of minimising cross-reactivity whilst improving specificity and sensitivity [36, 68, 69]. Furthermore, *in silico* discovery of immunoreactive B cell epitopes may allow the design of species-specific epitopes for serological diagnosis. Moreover, the possibility of construction of multi-epitopic chimeric proteins could improve the diagnostic accuracy of synthetic peptides [37, 70].

In the present review, we identified two studies [16, 37] that investigated peptide based serum IgG ELISA and one study [36] that utilized a multi-peptide chimeric protein. Except for Smp_167240 (213–228), Smp_141860 (1694–1709), Smp_141860 (1694–1706) and Sm 156860 which had poor diagnostic performances most peptides identified in this review had good diagnostic performances with sensitivities ranging from 67.71% to 96.15%, specificities ranging from 69.23% to 100% and AUC ranging from 0.76 (95% CI 0.6024–0.9213) to 0.99 (95% CI 0.987–100) (Table 4).

**Table 4:** Diagnostic performance *S*. *mansoni* peptides and chimeric proteins antibody detection tests.

Peptide/Antigen	Peptide sequence	Serological assay	Sensitivity	Specificity	AUC& (95% CI)	Reference
Smp_136560 (1564–1578)	ITEGNNSREGNSEKV	Serum IgG ELISA	73.08%	76.92%	0.76 (0.6024–0.9213)	Gardenia B. F. Carvalho [16]
Smp_141860 (1694–1709)	NHSMDKDDDDFSDIDK	Serum IgG ELISA	84.62%	46.15%	0.58 (0.316–0.8041)	
Smp_093840(219–233)	TTTNKDDTQINSAPS	Serum IgG ELISA	69.23%	76.92%	0.78 (0.6277–0.9374))	
Smp_126160(438–452)	LVTPESKYYSSLPGN	Serum IgG ELISA	84.62%	69.23%	0.82 (0.6871–0.9609)	
Smp_150390.1(216–230)	SLPSNAHNNDNNSSD	Serum IgG ELISA	96.15%	100%	0.99 (0.9874–1.00)	
Smp_167240(213–228)	QCDLDTQWNPAGTEYS	Serum IgG ELISA	53.85%	53.85%	0.51(0.3068–0.7198)	
Smp_180240(339–353)	RDWPTTLTGAGGSTT	Serum IgG ELISA	73.08%	92.31%	0.87 (0.7633–0.9823)	
Sm041370	QVFYYKDMYSLWLADT	Serum IgG ELISA	88.24%	100%	-	Marcelo D. Lopes [37]
Sm140560	MGFATGYFVHDIYHNI	Serum IgG ELISA	70.59%	91.67%	-	
Sm156860	QLCKDTSSRMKAAFQF	Serum IgG ELISA	47.06%	91.67%	-	
Sm168240	HTLVKSRRLNGTIEFP	Serum IgG ELISA	64.71%	83.33%	-	
Chimeric protein (Cathepsin B antigen and ET03 clone peptides chimeric protein)	Sm 31_81–100, Sm31_221–240, Sm 31_241–260, ET03_5–24 and ET03_34–53	Serum IgG ELISA	86.8% (95% CI 74.1–94%)	94.2% (95% CI 85–97.3%)	-	E. De Oliveiraa [36]

Oliveira and colleagues used a chimeric protein from a pool of 5 epitopes derived from *S*. *mansoni* proteins in a serum based IgG ELISA and demonstrated high diagnostic performances. The chimeric had a sensitivity of 86.8% (95% CI 74.1%-94%) and a specific of 94.2% (95% CI 85%-97.3%). They further explained that the high specificity was due to low cross-reactivity of the *S*. *mansoni* chemically synthesised immunodominant peptides with sera from non-infected individuals. They went on to explain that diagnostic sensitivity of the chimeric protein may be improved by the inclusion of other peptides, originating from the same or different proteins [36].

## Conclusion

This review identifies recombinant antigens that were grouped into eleven schistosome protein families’ viz., SERPIN, Pur DNA binding protein, MEC 2, tetraspanin, peptidases, ferritins and haemoglobinase for *S*. *haematobium*. Cheparones, MEGs proteins, SAPLIP and calreticulin were identified for *S*. *mansoni*. The tetraspanin CD63 antigen had the best overall performance for *S*. *haematobium* diagnosis. The tetraspanin CD63 antigen Serum IgG POC-ICTs had a sensitivity of 89% and a specificity of 100%. Peptide Smp_150390.1 (216–230) serum based IgG ELISA had the best overall performance for *S*. *mansoni* diagnosis with a sensitivity of 96.15%, specificity of 100% and an AUC of 0.99. Synthetic peptides were reported to demonstrate good to excellent diagnostic performances. The possibility of construction of multi-peptide chimeric proteins could further improve the diagnostic accuracy of synthetic peptides. Together with the advantages associated with urine sampling technique due to its non-invasive nature, we recommend development of multi-peptide chimeric proteins urine based point of care tools that can be used in remote settings.

## Supporting information

S1 File Search strategy and results for all databases. (PDF)

S1 Table PRISMA-P checklist. (PDF)

S2 Table Studies included and excluded from the review after full text screening and reasons for exclusion. (XLSX)
