# Supplementary material for: Diagnostic performances of Schistosoma haematobium and Schistosoma mansoni recombinant proteins, peptides and chimeric proteins antibody based tests. Systematic scoping review
Source: PLoS One. 2023 Mar 2;18(3):e0282233. doi: 10.1371/journal.pone.0282233 (PMC9980832; doi:10.1371/journal.pone.0282233)
Supplement: S1 File — (PDF) [file pone.0282233.s001.pdf]

Search Strategy

Schistosomiasis or bilharzia or snail fever or schistosome or schistosoma or S. haematobium or S. mansoni or bilharziasis or katayama fever or Bilharziase.

AND

Peptides or Epitopes or antigens or proteins or polypeptides or antigenic determinant

PubMed Search Results (February 14 2022)

Query	Results
(Schistosomiasis[Title/Abstract] OR bilharzia[Title/Abstract] OR snail fever[Title/Abstract] OR schistosome[Title/Abstract] OR schistosoma[Title/Abstract] OR S. haematobium[Title/Abstract] OR S. mansoni[Title/Abstract] OR bilharziasis[Title/Abstract] OR katayama fever[Title/Abstract] OR Bilharziase[Title/Abstract]) AND (Peptides[Title/Abstract] OR Epitopes[Title/Abstract] OR antigens[Title/Abstract] OR proteins[Title/Abstract] OR polypeptides[Title/Abstract] OR antigenic determinant[Title/Abstract])	3,893
Peptides[Title/Abstract] OR Epitopes[Title/Abstract] OR antigens[Title/Abstract] OR proteins[Title/Abstract] OR polypeptides[Title/Abstract] OR antigenic determinant[Title/Abstract]	1,671,179
Schistosomiasis[Title/Abstract] OR bilharzia[Title/Abstract] OR snail fever[Title/Abstract] OR schistosome[Title/Abstract] OR schistosoma[Title/Abstract] OR S. haematobium[Title/Abstract] OR S. mansoni[Title/Abstract] OR bilharziasis[Title/Abstract] OR katayama fever[Title/Abstract] OR Bilharziase[Title/Abstract]	32,382

CINAHL (February 16 2022)

#	Query	Limiters/Expanders	Last Run Via	Results
S21	S11 AND S20	Search modes - Find all my search terms	Interface - EBSCOhost Research Databases<br>Search Screen - Advanced Search<br>Database - CINAHL	143
S20	S13 OR S14 OR S15 OR S16 OR S17 OR S18 OR S19	Search modes - Find all my search terms	Interface - EBSCOhost Research Databases<br>Search Screen - Advanced Search<br>Database - CINAHL	180,318
S19	AB Antigenic determinants	Search modes - Find all my search terms	Interface - EBSCOhost Research Databases<br>Search Screen - Advanced Search<br>Database - CINAHL	75
S18	AB Polypeptides	Search modes - Find all my search terms	Interface - EBSCOhost Research Databases<br>Search Screen - Advanced Search<br>Database - CINAHL	1,953
S17	AB Recombinant protein	Search modes - Find all my search terms	Interface - EBSCOhost Research Databases<br>Search Screen - Advanced Search<br>Database - CINAHL	3,772
S16	AB Recombinant antigen	Search modes - Find all my search terms	Interface - EBSCOhost Research Databases<br>Search Screen - Advanced Search<br>Database - CINAHL	959
S15	AB Proteins	Search modes - Find all my search terms	Interface - EBSCOhost Research Databases	153,202

			Search Screen - Advanced Search Database - CINAHL	
S14	AB Antigens	Search modes - Find all my search terms	Interface - EBSCOhost Research Databases Search Screen - Advanced Search Database - CINAHL	30,058
S13	AB Epitopes	Search modes - Find all my search terms	Interface - EBSCOhost Research Databases Search Screen - Advanced Search Database - CINAHL	2,457
S12	AB Peptides	Search modes - Find all my search terms	Interface - EBSCOhost Research Databases Search Screen - Advanced Search Database - CINAHL	23,650
S11	S1 OR S2 OR S3 OR S4 OR S5 OR S6 OR S7 OR S8 OR S9 OR S10	Search modes - Find all my search terms	Interface - EBSCOhost Research Databases Search Screen - Advanced Search Database - CINAHL	1,012
S10	AB Katayama fever	Search modes - Find all my search terms	Interface - EBSCOhost Research Databases Search Screen - Advanced Search Database - CINAHL	4
S9	AB Bilharziase	Search modes - Find all my search terms	Interface - EBSCOhost Research Databases Search Screen - Advanced Search Database - CINAHL	0
S8	AB Bilharziasis	Search modes - Find all my search terms	Interface - EBSCOhost Research Databases Search Screen - Advanced Search Database - CINAHL	17
S7	AB S. mansoni	Search modes - Find all my search terms	Interface - EBSCOhost Research Databases Search Screen - Advanced Search Database - CINAHL	202
S6	AB S. haematobium	Search modes - Find all my search terms	Interface - EBSCOhost Research Databases Search Screen - Advanced Search Database - CINAHL	122
S5	AB Schistosoma	Search modes - Find all my search terms	Interface - EBSCOhost Research Databases Search Screen - Advanced Search Database - CINAHL	532
S4	AB Schistosome	Search modes - Find all my search terms	Interface - EBSCOhost Research Databases Search Screen - Advanced Search Database - CINAHL	145
S3	AB Snail fever	Search modes - Find all my search terms	Interface - EBSCOhost Research Databases Search Screen - Advanced Search Database - CINAHL	6
S2	AB Bilharzia	Search modes - Find all my search terms	Interface - EBSCOhost Research Databases Search Screen - Advanced Search Database - CINAHL	25
S1	AB Schistosomiasis	Search modes - Find all my search terms	Interface - EBSCOhost Research Databases Search Screen - Advanced Search Database - CINAHL	778

Cochrane Library (February 20 2022)

105 Trials matching Schistosomiasis or bilharzia or snail fever or schistosome or schistosoma or S. haematobium or S. mansoni or bilharziasis or katayama fever or Bilharziase. in Title Abstract Keyword AND Peptides or Epitopes or antigens or proteins or polypeptides or antigenic determinant in Title Abstract Keyword - (Word variations have been searched)

[Cochrane Central Register of Controlled Trials]

Issue 2 of 12, February 2022

105 Trials matching **Schistosomiasis or bilharzia or snail fever or schistosome or schistosoma or S. haematobium or S. mansoni or bilharziasis or katayama fever or Bilharziase. in Title Abstract Keyword AND Peptides or Epitopes or antigens or proteins or polypeptides or antigenic determinant in Title Abstract Keyword - (Word variations have been searched)**

PsycInfo (February 20 2022)

#	Query	Limiters/Expanders	Last Run Via	Results
S21	S11 AND S20	Search modes - Find all my search terms	Interface - EBSCOhost Research Databases<br>Search Screen - Advanced Search<br>Database - APA PsycInfo	16
S20	S12 OR S13 OR S14 OR S15 OR S16 OR S17 OR S18 OR S19	Search modes - Find all my search terms	Interface - EBSCOhost Research Databases<br>Search Screen - Advanced Search<br>Database - APA PsycInfo	96,649
S19	AB antigenic determinant	Search modes - Boolean/Phrase	Interface - EBSCOhost Research Databases<br>Search Screen - Advanced Search<br>Database - APA PsycInfo	9
S18	AB recombinants protein	Search modes - Boolean/Phrase	Interface - EBSCOhost Research Databases<br>Search Screen - Advanced Search<br>Database - APA PsycInfo	337
S17	AB polypeptides	Search modes - Boolean/Phrase	Interface - EBSCOhost Research Databases<br>Search Screen - Advanced Search<br>Database - APA PsycInfo	1,300
S16	AB Proteins	Search modes - Boolean/Phrase	Interface - EBSCOhost Research Databases<br>Search Screen - Advanced Search<br>Database - APA PsycInfo	81,707
S15	AB Antigens	Search modes - Boolean/Phrase	Interface - EBSCOhost Research Databases<br>Search Screen - Advanced Search<br>Database - APA PsycInfo	4,623

S14	AB Epitome	Search modes - Boolean/Phrase	Interface - EBSCOhost Research Databases<br>Search Screen - Advanced Search<br>Database - APA PsycInfo	242
S13	AB Epitopes	Search modes - Boolean/Phrase	Interface - EBSCOhost Research Databases<br>Search Screen - Advanced Search<br>Database - APA PsycInfo	893
S12	AB Peptides	Search modes - Boolean/Phrase	Interface - EBSCOhost Research Databases<br>Search Screen - Advanced Search<br>Database - APA PsycInfo	15,759
S11	S1 OR S2 OR S3 OR S4 OR S5 OR S6 OR S7 OR S8 OR S9 OR S10	Search modes - Find all my search terms	Interface - EBSCOhost Research Databases<br>Search Screen - Advanced Search<br>Database - APA PsycInfo	150
S10	AB Katayama fever	Search modes - SmartText Searching	Interface - EBSCOhost Research Databases<br>Search Screen - Advanced Search<br>Database - APA PsycInfo	1
S9	AB Katayama fever	Search modes - Find all my search terms	Interface - EBSCOhost Research Databases<br>Search Screen - Advanced Search<br>Database - APA PsycInfo	0
S8	AB Bilharziasis	Search modes - Find all my search terms	Interface - EBSCOhost Research Databases<br>Search Screen - Advanced Search<br>Database - APA PsycInfo	1
S7	AB S. mansoni	Search modes - Find all my search terms	Interface - EBSCOhost Research Databases<br>Search Screen - Advanced Search<br>Database - APA PsycInfo	16
S6	AB S. haematobium	Search modes - Find all my search terms	Interface - EBSCOhost Research Databases<br>Search Screen - Advanced Search<br>Database - APA PsycInfo	5
S5	AB schistosome	Search modes - Find all my search terms	Interface - EBSCOhost Research Databases<br>Search Screen - Advanced Search<br>Database - APA PsycInfo	11
S4	AB schistosoma	Search modes - Find all my search terms	Interface - EBSCOhost Research Databases<br>Search Screen - Advanced Search<br>Database - APA PsycInfo	42
S3	AB Snail fever	Search modes - Find all my search terms	Interface - EBSCOhost Research Databases<br>Search Screen - Advanced Search<br>Database - APA PsycInfo	1

S2	AB bilharzia	Search modes - Find all my search terms	Interface - EBSCOhost Research Databases<br>Search Screen - Advanced Search<br>Database - APA PsycInfo	1
S1	AB Schistosomiasis	Search modes - Find all my search terms	Interface - EBSCOhost Research Databases<br>Search Screen - Advanced Search<br>Database - APA PsycInfo	96

EMBASE

**20**	**limit 19 to yr="2000 - 2022"**	**2239**	**Type**
**19**	**10 and 18**	**3839**	**Advanced**
**18**	**11 or 12 or 13 or 14 or 15 or 16 or 17**	**1810167**	**Advanced**
**17**	**antigenic determinants.ab.**	**6324**	**Advanced**
**16**	**recombinant proteins.ab.**	**16364**	**Advanced**
**15**	**polypeptides.ab.**	**38960**	**Advanced**
**14**	**proteins.ab.**	**1376927**	**Advanced**
**13**	**Antigens.ab.**	**227996**	**Advanced**
**12**	**Epitopes.ab.**	**62297**	**Advanced**
**11**	**Peptides.ab.**	**261362**	**Advanced**
**10**	**1 or 2 or 3 or 4 or 5 or 6 or 7 or 8 or 9**	**25428**	**Advanced**
**9**	**Katayama fever.ab.**	**39**	**Advanced**
**8**	**Bilharziasis.ab.**	**446**	**Advanced**
**7**	**S mansoni.ab.**	**6163**	**Advanced**
**6**	**S haematobium.ab.**	**2137**	**Advanced**
**5**	**Schistosoma.ab.**	**16246**	**Advanced**
**4**	**Schistosome.ab.**	**4700**	**Advanced**
**3**	**Snail fever.ab.**	**14**	**Advanced**
**2**	**Bilharzia.ab.**	**272**	**Advanced**
**1**	**Schistosomiasis.ab.**	**14550**	**Advanced**
